# Supplementary material for: Knowledge, attitude, and practice regarding COVID-19 outbreak in Bangladesh: An online-based cross-sectional study
Source: PLoS One. 2020 Oct 9;15(10):e0239254. doi: 10.1371/journal.pone.0239254 (PMC7546509; doi:10.1371/journal.pone.0239254)
Supplement: Attachment — Submitted filename: PONE-D-20-16724_reviewer.pdf [file pone.0239254.s002.pdf]

PLOS ONE

Knowledge, attitude, and practice regarding COVID-19 outbreak in Bangladesh: An online-based cross-sectional study

--Manuscript Draft--

Manuscript Number:	PONE-D-20-16724
Article Type:	Research Article
Full Title:	Knowledge, attitude, and practice regarding COVID-19 outbreak in Bangladesh: An online-based cross-sectional study
Short Title:	KAP and Covid-19
Corresponding Author:	David Gozal<br>University of Missouri Health Care<br>Columbia, MO UNITED STATES
Keywords:	COVID-19; knowledge; attitude; perception; practice; pandemic; Bangladesh
Abstract:	In Bangladesh, an array of measures have been adopted to control the rapid spread of the COVID-19 epidemic. Such general population control measures could significantly influence perception, knowledge, attitudes, and practices (KAP) towards COVID-19. Here, we assessed KAP towards COVID-19 immediately after the lock-down measures were implemented and during the rapid rise period of the outbreak. Online-based cross-sectional study conducted from March 29 to April 19, 2020, involving Bangladeshi residents aged 12–64 years, recruited via social media. After consenting, participants completed an online survey assessing socio-demographic variables, perception, and KAP towards COVID-19. Of the 2017 survey participants, 59.8% were male, the majority were students (71.2%), aged 21-30 years (57.9%), having a bachelor's degree (61.0%), having family income >30,000 BDT (50.0%), and living in urban areas (69.8). The survey revealed that 48.3% of participants had more accurate knowledge, 62.3% had more positive attitudes, and 55.1% had more frequent practices regarding COVID-19 prevention. Majority (96.7%) of the participants agreed 'COVID-19 is a dangerous disease', almost all (98.7%) participants wore a face mask in crowded places, 98.8% agreed to report a suspected case to health authorities, and 93.8% implemented washing hands with soap and water. In multiple logistic regression analyses, COVID-19 more accurate knowledge was associated with age and residence. Sociodemographic factors such as being older, higher education, employment, monthly family income >30,000 BDT, and having more frequent prevention practices were the more positive attitude factors. More frequent prevention practice factors were associated with female sex, older age, higher education, family income > 30,000 BDT, urban area residence, and having more positive attitudes. To improve KAP of general populations is crucial during the rapid rise period of a pandemic outbreak such as COVID-19. Therefore, development of effective health education programs that incorporate considerations of KAP-modifying factors is needed.
Order of Authors:	Most Zannatul Ferdousi
	Md. Saiful Islam
	Md. Tajuddin Sikder
	Abu Syed Md. Mosaddek
	JA Zegarra-Valdivia
	David Gozal
Additional Information:	
Question	Response
Financial Disclosure<br><br>Enter a financial disclosure statement that	The authors received no specific funding for this work.

Powered by Editorial Manager® and ProduXion Manager® from Aries Systems Corporation

describes the sources of funding for the work included in this submission. Review the submission guidelines for detailed requirements. View published research articles from *PLOS ONE* for specific examples.<br><br>This statement is required for submission and **will appear in the published article** if the submission is accepted. Please make sure it is accurate.<br><br>**Unfunded studies**<br>Enter: *The author(s) received no specific funding for this work.*<br><br>**Funded studies**<br>Enter a statement with the following details:<br>• Initials of the authors who received each award<br>• Grant numbers awarded to each author<br>• The full name of each funder<br>• URL of each funder website<br>• Did the sponsors or funders play any role in the study design, data collection and analysis, decision to publish, or preparation of the manuscript?<br>• **NO** - Include this sentence at the end of your statement: *The funders had no role in study design, data collection and analysis, decision to publish, or preparation of the manuscript.*<br>• **YES** - Specify the role(s) played.<br><br>* typeset	
**Competing Interests**<br><br>Use the instructions below to enter a competing interest statement for this submission. On behalf of all authors, disclose any competing interests that could be perceived to bias this work—acknowledging all financial support and any other relevant financial or non-financial competing interests.<br><br>This statement **will appear in the published article** if the submission is accepted. Please make sure it is accurate. View published research articles from *PLOS ONE* for specific examples.	The authors have declared that no competing interests exist.

Powered by Editorial Manager® and ProduXion Manager® from Aries Systems Corporation

**NO authors have competing interests**<br><br>Enter: *The authors have declared that no competing interests exist.*<br><br>**Authors with competing interests**<br><br>Enter competing interest details beginning with this statement:<br><br>*I have read the journal's policy and the authors of this manuscript have the following competing interests: [insert competing interests here]*<br><br>* typeset	
**Ethics Statement**<br><br>Enter an ethics statement for this submission. This statement is required if the study involved:<br><br>• Human participants<br>• Human specimens or tissue<br>• Vertebrate animals or cephalopods<br>• Vertebrate embryos or tissues<br>• Field research<br><br>Write "N/A" if the submission does not require an ethics statement.<br><br>General guidance is provided below. Consult the submission guidelines for detailed instructions. **Make sure that all information entered here is included in the Methods section of the manuscript.**	This study was approved by the Institutional review Board at Jahangirnagar University

Powered by Editorial Manager® and ProduXion Manager® from Aries Systems Corporation

**Format for specific study types**<br><br>**Human Subject Research (involving human participants and/or tissue)**<br>• Give the name of the institutional review board or ethics committee that approved the study<br>• Include the approval number and/or a statement indicating approval of this research<br>• Indicate the form of consent obtained (written/oral) or the reason that consent was not obtained (e.g. the data were analyzed anonymously)<br><br>**Animal Research (involving vertebrate animals, embryos or tissues)**<br>• Provide the name of the Institutional Animal Care and Use Committee (IACUC) or other relevant ethics board that reviewed the study protocol, and indicate whether they approved this research or granted a formal waiver of ethical approval<br>• Include an approval number if one was obtained<br>• If the study involved *non-human primates*, add *additional details* about animal welfare and steps taken to ameliorate suffering<br>• If anesthesia, euthanasia, or any kind of animal sacrifice is part of the study, include briefly which substances and/or methods were applied<br><br>**Field Research**<br><br>Include the following details if this study involves the collection of plant, animal, or other materials from a natural setting:<br>• Field permit number<br>• Name of the institution or relevant body that granted permission	
**Data Availability**<br><br>Authors are required to make all data underlying the findings described fully available, without restriction, and from the time of publication. PLOS allows rare exceptions to address legal and ethical concerns. See the PLOS Data Policy and FAQ for detailed information.	Yes - all data are fully available without restriction

A Data Availability Statement describing where the data can be found is required at submission. Your answers to this question constitute the Data Availability Statement and **will be published in the article**, if accepted.<br><br>**Important:** Stating 'data available on request from the author' is not sufficient. If your data are only available upon request, select 'No' for the first question and explain your exceptional situation in the text box.<br><br>Do the authors confirm that all data underlying the findings described in their manuscript are fully available without restriction?	
**Describe where the data may be found in full sentences. If you are copying our sample text, replace any instances of XXX with the appropriate details.**<br><br>• If the data are **held or will be held in a public repository**, include URLs, accession numbers or DOIs. If this information will only be available after acceptance, indicate this by ticking the box below. For example: *All XXX files are available from the XXX database (accession number(s) XXX, XXX.).*<br>• If the data are all contained **within the manuscript and/or Supporting Information files**, enter the following: *All relevant data are within the manuscript and its Supporting Information files.*<br>• If neither of these applies but you are able to provide **details of access elsewhere**, with or without limitations, please do so. For example:<br><br>*Data cannot be shared publicly because of [XXX]. Data are available from the XXX Institutional Data Access / Ethics Committee (contact via XXX) for researchers who meet the criteria for access to confidential data.*<br><br>*The data underlying the results presented in the study are available from (include the name of the third party*	all data will be available from the authors upon request.

and contact information or URL).<br>• This text is appropriate if the data are owned by a third party and authors do not have permission to share the data.<br><br>* typeset	
Additional data availability information:	Tick here if your circumstances are not covered by the questions above and you need the journal's help to make your data available.

Powered by Editorial Manager® and ProduXion Manager® from Aries Systems Corporation

Cover Letter

Click here to access/download;Manuscript;Manuscript PLOS One.docx

Knowledge, attitude, and practice regarding COVID-19 outbreak in Bangladesh: An online-based cross-sectional study

Most. Zannatul Ferdous[a,d], Md. Saiful Islam[a,b], Md. Tajuddin Sikder[a], Abu Syed Md. Mosaddek[c,d], J. A. Zegarra-Valdivia[e], David Gozal[f,*]

[a] Department of Public Health and Informatics, Jahangirnagar University, Savar, Dhaka-1342, Bangladesh

[b] Youth Research Association, Dhaka, Bangladesh

[c] Department of Pharmacology, Uttara Adhunik Medical College, Uttara, Dhaka-1230, Dhaka, Bangladesh

[d] Clinical Affairs, Quest Bangladesh

[e] Universidad Nacional de San Agustín de Arequipa, Perú

[f] Department of Child Health and the Child Health Research Institute, The University of Missouri School of Medicine, Columbia, MO 65201, USA (DG)

*** Corresponding Author**

E-mail: gozald@health.missouri.edu (DG)

Authors contributions

MSI: Conceptualization, Methodology, Investigation, Data curation, Formal analysis, Writing-original draft, Editing, Validation., MZF: Conceptualization, Investigation, Writing-original draft, Editing, Validation. MTS: Editing, Validation., ASMM: Editing, Validation., JAZV: Editing, Validation., DG: Editing, Validation.

Abstract

In Bangladesh, an array of measures have been adopted to control the rapid spread of the COVID-19 epidemic. Such general population control measures could significantly influence perception, knowledge, attitudes, and practices (KAP) towards COVID-19. Here, we assessed KAP towards COVID-19 immediately after the lock-down measures were implemented and during the rapid rise period of the outbreak. Online-based cross-sectional study conducted from March 29 to April 19, 2020, involving Bangladeshi residents aged 12–64 years, recruited via social media. After consenting, participants completed an online survey assessing socio-demographic variables, perception, and KAP towards COVID-19. Of the 2017 survey participants, 59.8% were male, the majority were students (71.2%), aged 21-30 years (57.9%), having a bachelor's degree (61.0%), having family income >30,000 BDT (50.0%), and living in urban areas (69.8). The survey revealed that 48.3% of participants had more accurate knowledge, 62.3% had more positive attitudes, and 55.1% had more frequent practices regarding COVID-19 prevention. Majority (96.7%) of the participants agreed 'COVID-19 is a dangerous disease', almost all (98.7%) participants wore a face mask in crowded places, 98.8% agreed to report a suspected case to health authorities, and 93.8% implemented washing hands with soap and water. In multiple logistic regression analyses, COVID-19 more accurate knowledge was associated with age and residence. Sociodemographic factors such as being older, higher education, employment, monthly family income >30,000 BDT, and having more frequent prevention practices were the more positive attitude factors. More frequent prevention practice factors were associated with female sex, older age, higher education, family income > 30,000 BDT, urban area residence, and having more positive attitudes. To improve KAP of general populations is crucial during the rapid rise period of a pandemic outbreak such as COVID-19. Therefore, development of effective health education programs that incorporate considerations of KAP-modifying factors is needed.

Introduction

Coronavirus disease 2019 (COVID -19) is a global public health threat and has evolved to become a pandemic crisis around the world, which is caused by the severe acute respiratory syndrome, coronavirus 2 (SARS-CoV-2) [1]. In response to this serious situation, COVID-19 was declared as a public health emergency of international concern by the World Health Organization (WHO) on January 30 and called for collaborative efforts of all countries to prevent the rapid spread of COVID-19 [2].In Bangladesh, the first confirmed case was reported on 8 March 2020 [3]. Infection rates apparently remained low until the end of March, but a steep rise in cases began in April 2020 with case doubling times of 2 days [4]. As of 01 June 2020, according to the Institute of Epidemiology, Disease Control and Research (IEDCR), in Bangladesh 49,534 confirmed cases were reported, including 10,597 (21.4%) who recovered, and 672 (1.36%) related deaths [3]. The highest attack rate (AR) was observed in Dhaka city (874.9/1,000,000), followed by (2,040/1,000,000), followed by Narayanganj district (616.2/1,000,000), Munshiganj (432.4/1,000,000), Gazipur (168.7/1,000,000), Gopalganj (145.7/1,000,000), [3].

COVID-19 prompted implementation of public health protocols to control the spread of the virus, many of them involving social distancing, hand washing, and lockdown procedures, but has also resulted in creating public anguish and massive fear [5], particularly among the unaffected population [6]. Bangladesh has not previously experienced epidemics such as SARS or MERS, and it is clear that the public healthcare systems are not readily prepared for COVID-19. The magnitude and rapid proliferation of COVID-19 through slightly symptomatic or asymptomatic infected people in Bangladesh stresses the need to identify the behavioral responses of the population, such as to better address behavioral determinants of pandemic control [7]. Official measures such as school closures, shutdown of offices for an initial 30-day

duration, restrictions on leaving home after 6.00 pm, and legal actions on individuals leaving their dwellings after 7.00 pm, along with gathering restrictions in mosques and people gatherings have rapidly been imposed in many regions of the country [8,9]. However, for such measures to be effective, public adherence is essential, which is affected by their knowledge, attitudes, and practices (KAP) towards COVID-19 [10,11]. There are a limited number of studies on knowledge and attitudes during epidemics that have been conducted in Bangladesh. However, the lessons learned from the studies conducted in other countries in an epidemic situation such as the SARS outbreak in 2003 suggest that knowledge and attitudes towards infectious diseases are associated with serious panic and other emotional reactions among the population, which can further complicate attempts to prevent the spread of the disease [12, 13]. Suggestions from a Latin America-based study during the outbreaks of chikungunya, zika, and dengue reported low levels of participation and commitment to the imposed control measures in populations [14].

KAP is an important cognitive key in public health regarding health prevention and promotion. It involves a range of beliefs about the causes of the disease and exacerbating factors, identification of symptoms, and available methods of treatments and consequences [15]. Beliefs about COVID-19 come from different sources, such as stereotypes concerning similar viral diseases, governmental information, social media and internet, previous personal experiences, and medical sources. The accuracy of these beliefs may determine different behaviors about prevention and could vary in the population. In many cases, the absence of knowledge, or if most of these medical-related beliefs are actually false may carry a potential risk factor [16]. In Hubei, China, one of the first studies analyzing attitudes and knowledge about COVID-19 concluded that attitudes towards government measures to contain the epidemic were highly associated with the level of knowledge about COVID-19 [17]. The

authors reported that higher levels of information and education were associated with more positive attitudes towards COVID-19 preventive practices [5,17]. Perception of risk is also a key factor in commitment to prevention during outbreaks of global epidemics [5, 18-21].

Considering the lack of studies related to coronavirus epidemics and how to facilitate outbreak management of COVID-19 in Bangladesh, there is an urgent need to understand the public's KAP of COVID-19. Here, we aimed to investigate KAP towards COVID-19 during the rapid rise period and immediately after the implementation of lockdown measures in Bangladesh.

Methods

Participation and procedure

A cross-sectional and anonymous online population-based survey was conducted among individuals aged 12–64 years. The survey was conducted from March 29 to April 20, 2020, immediately after the implementation of lockdown measures by the government of Bangladesh. A semi-structured questionnaire was designed for the Google survey tool (Google Forms), and the generated link was shared to public on social media (i.e., Facebook, WhatsApp). The link was also shared personally to the contact list of investigators and research assistants. The investigators' decision to collect the data using online approaches was predicated on maintaining social distance during the strict lockdown in Bangladesh. Initially, 2,068 potential respondents provided informed consent. Of these, 2,017 respondents completed the entire survey, generating a response rate of 97.5%. The inclusion criteria to participate in the study were being a Bangladeshi resident, having internet access, and volunteering.

voluntary participation?

Measures

A semi-structured and self-reported questionnaire containing informed consent, questions regarding socio-demographics, knowledge, attitude, and practice.

Socio-demographic measures

Socio-demographic information was collected, including gender, age, education, occupation, marital status, nature of the family (nuclear/joint, with the joint being an extended family, often of multiple generations), number of family members, monthly family income, and location of permanent residence.

Knowledge, attitude, and practice

To assess the level of knowledge, attitude, and practice of the respondents, a total of 17 questions (including 6 for knowledge, 6 for attitude, and 7 for practice) were included. The survey questions were adapted and modified from previously published literature regarding viral epidemics related to MERS-CoV disease [23,24], infection prevention and control measures for COVID-19 by World Health Organization [25], and guidelines suggested by the country's Institute of Epidemiology, Disease Control and Research (IEDCR) [26].

After completion of the initial draft of the survey questionnaire, it was validated and adopted as follows: Firstly, the questionnaire was sent to four experts. After coordination and consensus of all experts' opinions, the final questionnaire was drafted, and underwent pilot testing in 30 individuals to confirm the reliability of the questionnaire. The data from the pilot study were loaded into SPSS version 25, and subjected to reliability coefficient analysis. The Cronbach's alpha coefficient of the KAP questions was 0.73, which indicates acceptable internal consistency [27].

The knowledge section consisted of 6 items of a and each question was responded as "*Yes*", "*No*" and "*Don't know*". The correct answer was coded as 1, while the wrong answer was coded as 0. The total score ranged from 0-6, with an overall greater score indicates more accurate knowledge. A cut off level of ≥4 was set for more accurate knowledge.

The attitude section consisted of 6 items, and the response of each item was responded on a 3-point Likert scale as follows 0 ("*Disagree*"), 1 ("*Undecided*"), and 2("*Agree*"). The total score ranges from 0 to 12, with an overall greater score indicates more positive attitudes towards ~~the~~ COVID-19. A cut off level of ≥11was set for more positive attitudes towards the prevention of COVID-19.

The practice section included 7 items practice measures responding to the COVID-19, and each item was responded as "*Yes*", "*No*", and "*Sometimes*". Practice items' total score ranges from 0-7, with an overall greater score indicates more frequent practices towards the COVID-19. A cut off level of ≥6was set for more frequent practices.

Statistical analysis

The data analysis was performed using Microsoft Excel 2019 and SPSS version 25.0 (Chicago, IL, USA). Microsoft Excel was used for editing, sorting, and coding. The excel file was then imported into SPSS software. Descriptive statistics (frequencies, percentages, means, standard deviations) and first-order analyses (i.e., chi-square tests) were performed. Binary logistic regression was performed with a 95% confidence interval to determine significant associations between categorical dependent and independent variables.

Ethical considerations

The study was conducted in accordance with the Institutional Research Ethics and the declaration of Helsinki. Formal ethical approval was granted by the Ethical Review Committee, Uttara Adhunik Medical College, Uttara, Dhaka-1260, Bangladesh (Ref: UAMC/ERC/04/2020). The consent form documented the aims, nature, and procedure of the study. Anonymity and confidentially were strictly maintained.

Results

A total of 2,017 respondents were included in the final analysis, of which 59.8% male with an average age was 24.4±5.4 years (SD) ranging from 12 to 64 years. Almost all respondents were not married (80.8%). The majority were students (71.2%), had a bachelor's level of education (61.0%), came from urban areas (69.8%), lived in nuclear families (77.9%) and their monthly family income was >30,000 BDT (50.0%) (Table 1).

Table 1. Demographic characteristics of participants (N=2,017).

Variables	n	(%)
**Gender**		
Male	1206	(59.8)
Female	811	(40.2)
**Age**		
12-20	671	(33.3)
21-30	1168	(57.9)
>30	178	(8.8)
**Education**		
Secondary (6-10)	20	(1.0)
Intermediate (11-12)	226	(11.2)
Bachelor	1230	(61.0)
Higher education (above bachelor)	541	(26.8)
**Marital status**		
Unmarried	1630	(80.8)
Married	379	(18.8)
Divorced	8	(0.4)
**Occupation**		
Student	1437	(71.2)
Housewife	64	(3.2)
Govt. employee	122	(6.0)
Non-govt. employee	315	(15.6)

Businessman	52	(2.6)
Unemployment	27	(1.3)
**Family type**		
Nuclear	1572	(77.9)
Join	445	(22.1)
**No of family member**		
<5	1449	(71.8)
≥5	568	(28.2)
**Monthly family income**		
<20,000	512	(25.4)
20,000-30,000	497	(24.6)
>30,000	1008	(50.0)
**Residence**		
Rural area	610	(30.2)
Urban area	1407	(69.8)

Perception towards the COVID-19 about mode of transmission, incubation period, symptoms, risk factors, treatments, prevention, initiatives, and challenges

In the perception component, Table 2 depicts our findings. For the mode of transmission, more than half of the respondents reported close contact with an infected person (93.7%), direct transmission during coughing (66.4%), touching contaminated surfaces (61.3%), along with others as just as contact with infected animals (30.8%), through eating infected animal products (e.g., meat, milk) (21.4%), and only 0.5% had no idea about the mode of transmission of COVID-19. Most of the respondents (91.3%) reported the correct incubation period (2-14 days), and only 2.4% had no knowledge. Most of the respondents (99.4%) reported fever, dry cough, and difficulty breathing as the common symptoms of the COVID-19. On the other hand, half of the respondents (51.2%) reported sore throat, nasal stuffiness, along with headache (0.1%), diarrhea (0.7%), and no idea (0.4%).

Table 2. Perception towards COVID-19 about the mode of transmission, incubation period, symptoms, risk factors, treatments, prevention initiatives, and challenges.

Variables	Total N=2017		Male		Female	
	n	(%)	n	(%)	n	(%)
**How is COVID-19 spread?**[a]						
Direct transmission during coughing	1339	(66.4)	816	(60.9)	523	(39.1)
Touching contaminated surfaces	1236	(61.3)	773	(62.5)	463	(37.5)
Contact with infected animals	622	(30.8)	396	(63.7)	226	(36.3)
Through eating infected animal products (e.g., meat, milk)	431	(21.4)	276	(64.0)	155	(36.0)
Close contact with an infected person	1889	(93.7)	1129	(59.8)	760	(40.2)
Don't know	11	(0.5)	7	(63.6)	4	(36.4)
**Symptoms appear after which of the following?**						
2-5 days	126	(6.2)	82	(65.1)	44	(34.9)
2-14 days	1842	(91.3)	1092	(59.3)	750	(40.7)
Don't know	49	(2.4)	32	(65.3)	17	(34.7)
**What are the symptoms of COVID-19?**[a]						
Fever, dry cough, difficulty of breathing	2004	(99.4)	1199	(59.8)	805	(40.2)
Sore throat, blocked nose	1032	(51.2)	614	(59.5)	418	(40.5)
Headache	3	(0.1)	0	(0.0)	3	(100.0)
Diarrhea	14	(0.7)	10	(71.4)	4	(28.6)
Don't know	8	(0.4)	5	(62.5)	3	(37.5)
**Who is most at risk for COVID-19 infection?**[a]						
Old aged persons	1737	(86.1)	1056	(60.8)	681	(39.2)
Pregnant women	427	(21.2)	249	(58.3)	178	(41.7)
Children	511	(25.3)	305	(59.7)	206	(40.3)
Individuals with cancer, diabetes, chronic respiratory diseases	1505	(74.6)	878	(58.3)	627	(41.7)
Migrants from other parts of the world having COVID-19	903	(44.8)	525	(58.1)	378	(41.9)
Don't know	17	(0.8)	15	(88.2)	2	(11.8)
**Which of the following describes COVID-19 treatment?**						
Supportive treatment	1627	(80.7)	967	(59.4)	660	(40.6)
Vaccine	20	(1.0)	11	(55.0)	9	(45.0)
Don't know	370	(18.3)	228	(61.6)	142	(38.4)
**What to do to prevent coronavirus?**[a]						
Wash hands with water and soap	1885	(93.5)	1136	(60.3)	749	(39.7)
Avoid touching the eyes andnose with hands	1823	(90.4)	1102	(60.4)	721	(39.6)
Avoid contacts with infected people	1709	(84.7)	1035	(60.6)	674	(39.4)
Using masks	1759	(87.2)	1054	(59.9)	705	(40.1)
Maintaining social distance	1886	(93.5)	1140	(60.4)	746	(39.6)
Maintaining self-quarantine	1551	(76.9)	945	(60.9)	606	(39.1)
Takingall family members in home quarantine	1575	(78.1)	931	(59.1)	644	(40.9)
Strengthening to health care	1283	(63.6)	765	(59.6)	518	(40.4)
Creating a strong voluntary force to fight against COVID-19	539	(26.7)	346	(64.2)	193	(35.8)
**Have you taken any initiative to protect your family members?**[a]						
Temporary closure of outside people coming inside the home	1769	(87.8)	1037	(58.6)	732	(41.4)
Arrange for handwashing with soap inside or outside the home	1723	(85.5)	1039	(60.3)	684	(39.7)
Wash hands with soap after touching pets	794	(39.4)	501	(63.1)	293	(36.9)
**Have you faced any problems to create awareness in your family about COVID-19?**[a]						
Negligence about the severity of the disease	810	(40.3)	531	(65.6)	279	(34.4)
Reluctance to use masks	512	(25.5)	335	(65.4)	177	(34.6)
Not being able to stop going out of the house	1147	(57.1)	695	(60.6)	452	(39.4)
Don't face the problem	395	(19.7)	225	(57.0)	170	(43.0)

[a]indicates multiple responses.

The respondents identified risk groups for developing COVID-19 as follows: older age persons (86.1%), individuals with cancer, diabetes, chronic respiratory diseases (74.6%), migrants from other parts of the world having COVID-19 (44.8%), children (25.3%), pregnant women (21.2%), and no idea (0.8%). The majority (80.7%) reported supportive treatments, but a vaccine was rarely mentioned (1.0%), and 18.3% had no idea about the treatment options of COVID-19.

The respondents recognized the following preventive measures for the COVID-19: washing hands with water and soap (93.5%), maintaining social distance (93.5%), avoid touching the eyes, nose with hands (90.4%), using a mask (87.2%), avoid contacts with infected people (84.7%), taking all family members into home quarantine (78.1%), maintaining self-quarantine (76.9%), strengthening to health care (63.6%), and creating a strong force to fight against COVID-19 (26.7%).

The respondents took the initiative to protect their family members: temporary and absolute restricted access to outside people coming inside the home (87.8%), arrange for handwashing with soap inside or outside the home (85.5%), and wash hands with soap after touching pets (39.4%). The respondents also reported that they faced many problems to create awareness among their family members: not being able to stop from leaving the house (57.1%), negligence about the severity of the disease (40.3%), reluctance to use masks (25.5%), and only 19.7% had no problems.

Knowledge

For each question of knowledge, the distribution of responses from participants is presented in Table 3 with gender differences. There were no significant gender differences for each item of

knowledge questions; 48.3% of respondents had more accurate knowledge, and 51.7% of respondents had comparatively inaccurate knowledge regarding COVID-19. The rates of adequate knowledge were significantly more likely to be among (ⅰ)younger (12-20 years) (49.3% vs. 38.8% in aged more than 30 years, *p*=.029), and (ⅱ) be a respondent from a rural area (52.8% vs. 46.3% in those from an urban area, *p*=.008).

Table 3. Knowledge and gender difference of participants (N=2017).

**Variables**	Total N=2017		Male		Female		χ^2	df	*p*-value
	n	(%)	n	(%)	n	(%)			
**Is COVID-19 a dangerous disease?**									
Yes	1951	(96.7)	1160	(96.2)	791	(97.5)	2.988	2	0.224
No	40	(2.0)	27	(2.2)	13	(1.6)			
Don't know	26	(1.3)	19	(1.6)	7	(0.9)			
**Does it affect only humans?**									
Yes	1210	(60.0)	735	(60.9)	475	(58.6)	1.161	2	0.560
No	567	(28.1)	330	(27.4)	237	(29.2)			
Don't know	240	(11.9)	141	(11.7)	99	(12.2)			
**Does it transmit from humans to animals?**									
Yes	1013	(50.2)	612	(50.7)	401	(49.4)	4.690	2	0.096
No	578	(28.7)	358	(29.7)	220	(27.1)			
Don't know	426	(21.1)	236	(19.6)	190	(23.4)			
**Does it transmit from animals to humans?**									
Yes	1013	(50.2)	612	(50.7)	401	(49.4)	4.690	2	0.096
No	578	(28.7)	358	(29.7)	220	(27.1)			
Don't know	426	(21.1)	236	(19.6)	190	(23.4)			
**Is it transmitted by animal products (e.g., milk, meat)?**									
Yes	509	(25.2)	300	(24.9)	209	(25.8)	0.406	2	0.816
No	1017	(50.4)	615	(51.0)	402	(49.6)			
Don't know	491	(24.3)	291	(24.1)	200	(24.7)			
**Is it transmitted in well-cooked products?**									
Yes	53	(2.6)	35	(2.9)	18	(2.2)	3.859	2	0.145
No	1821	(90.3)	1076	(89.2)	745	(91.9)			
Don't know	143	(7.1)	95	(7.9)	48	(5.9)			

The sociodemographic factors of more accurate knowledge were 12-29 years age group vs. >30 years (OR=1.54; 95%CI=1.10-2.16, *p*=.012), and rural vs. urban areas (OR=1.295; 95CI%=1.07-1.57, *p*=.008).

Attitude

For each question focused on attitude, the distribution of responses from participants is presented in Table 4. The response rates of "*Agree*" were significantly higher in females

(99.5% vs. 98.3% in males, *p*=.043) to the item of attitude section regarding "*It is crucial to report a suspected case to health authorities*". Furthermore, the response rates of "*Agree*" were significantly higher in females (99.6% vs. 98.1% in males, *p*=.011) to the item ~~of attitude section regarding~~ "*It is important to use a face mask in a crowded place.*"

Table 4. Attitude and gender difference of participants (N=2017).

**Variables**	Total N=2017		Male		Female		χ^2	df	*p*-value
	n	(%)	n	(%)	n	(%)			
**It is crucial to report a suspected case to health authorities.**									
Agree	1993	(98.8)	1186	(98.3)	807	(99.5)	6.292	2	0.043
Undecided	12	(0.6)	11	(0.9)	1	(0.1)			
Disagree	12	(0.6)	9	(0.7)	3	(0.4)			
**It is important to use a face mask in crowded place.**									
Agree	1991	(98.7)	1183	(98.1)	808	(99.6)	9.053	2	0.011
Undecided	11	(0.5)	10	(0.8)	1	(0.1)			
Disagree	15	(0.7)	13	(1.1)	2	(0.2)			
**It is important to wash hands and face after coming outsides.**									
Agree	2006	(99.5)	1197	(99.3)	809	(99.8)	3.492	2	0.174
Undecided	5	(0.2)	5	(0.4)	0	(0.0)			
Disagree	6	(0.3)	4	(0.3)	2	(0.2)			
**COVID-19 is a preventable disease.**									
Agree	1829	(90.7)	1091	(90.5)	738	(91.0)	4.211	2	0.122
Undecided	109	(5.4)	60	(5.0)	49	(6.0)			
Disagree	79	(3.9)	55	(4.6)	24	(3.0)			
**It can be treated at home.**									
Agree	1158	(57.4)	686	(56.9)	472	(58.2)	1.995	2	0.369
Undecided	240	(11.9)	137	(11.4)	103	(12.7)			
Disagree	619	(30.7)	383	(31.8)	236	(29.1)			
**Health education can play an important role in COVID-19 prevention.**									
Agree	1928	(95.6)	1148	(95.2)	780	(96.2)	1.805	2	0.406
Undecided	52	(2.6)	32	(2.7)	20	(2.5)			
Disagree	37	(1.8)	26	(2.2)	11	(1.4)			

The findings indicated that 62.3% of respondents had more positive attitudes towards COVID-19. The rates of more positive attitudes were significantly more likely to be (i) among older individuals (> 30 years) (72.5% vs. 55.1% in aged 12-20 years, *p*<.001), and (ii) those with higher education (74.1% vs. 52.2% in intermediate [class 11-12], *p*<.001), (iii)married (70.4% vs. 37.5% in divorced, *p*=.001), (iv)housewives (78.1% vs. 58.2% in student, *p*<.001), (v) come from joint family (66.7% vs. 61.1% in nuclear family, *p*=.029), (vi) have monthly family income > 30,000 BDT (65.2% vs. 57.8% in those less than 20,000 BDT, *p*=.016), and (vii)have

more frequent safety-related preventive practices(66.1% vs. 57.7% in comparatively less frequent practices, $p<.001$).

Finally, regarding variables related to more positive attitudes against COVID-19, we found being younger (aged 12-20 years) vs. older (>30 years) (OR=0.47; 95%CI=0.33-.67, $p<.001$). Additional factors of more positive attitudes against COVID-19 were having higher education (above bachelor), being unemployed, having joint families, having monthly family income more than 30,000 BDT, and having more frequent practices (Table 6).

Practice

For each question-related to practice, the distribution of responses from participants is presented in Table 5. The response rates of "*Yes*" were significantly higher in females (81.8% vs. 73.5% in males, $p<.001$) to the item of practice section regarding "*Do you use tissues during coughing/sneezing?*", as well as "*Do you wash hands frequently using water and soaps?*" (95.6% vs. 92.5% in males, $p=.023$). Similarly, "Yes" response rates were significantly higher in females (96.2% vs. 87.1% in males, $p<.001$) to "*Do you maintain social distance (or home quarantine)?*", to *"Do you maintain a healthy lifestyle focusing on outbreak?*"(88.3% vs. 81.4% in males, $p<.001$), and to "*Do you obey all government rules related to the COVID-19?*" (93.0% vs. 85.4% in males, $p<.001$).

Table 5. Practice and gender difference of participants (N=2017).

**Variables**	Total N=2017		Male		Female		χ^2	df	*p*-value
	n	(%)	n	(%)	n	(%)			
**Do you use tissues or hanker chips during coughing/sneezing?**									
Yes	1549	(76.8)	886	(73.5)	663	(81.8)	23.392	2	<0.001
No	74	(3.7)	59	(4.9)	15	(1.8)			
Sometimes	394	(19.5)	261	(21.6)	133	(16.4)			
**Do you wash hands frequently using water and soaps?**									
Yes	1891	(93.8)	1116	(92.5)	775	(95.6)	7.570	2	0.023
No	14	(0.7)	10	(0.8)	4	(0.5)			
Sometimes	112	(5.6)	80	(6.6)	32	(3.9)			
**Do you avoid touching face and eyes?**									
Yes	1228	(60.9)	734	(60.9)	494	(60.9)	0.118	2	0.943

No	154	(7.6)	94	(7.8)	60	(7.4)			
Sometimes	635	(31.5)	378	(31.3)	257	(31.7)			
**Do you maintain social distance (or home quarantine)?**									
Yes	1831	(90.8)	1051	(87.1)	780	(96.2)	47.237	2	<0.001
No	41	(2.0)	34	(2.8)	7	(0.9)			
Sometimes	145	(7.2)	121	(10.0)	24	(3.0)			
**Do you eat healthy food focusing on outbreak?**									
Yes	760	(37.7)	464	(38.5)	296	(36.5)	0.806	1	0.369
No	1257	(62.3)	742	(61.5)	515	(63.5)			
Sometimes	0	(0.0)	0	(0.0)	0	(0.0)			
**Do you maintain a healthy lifestyle focusing on outbreak?**									
Yes	1698	(84.2)	982	(81.4)	716	(88.3)	17.779	2	<0.001
No	19	(0.9)	15	(1.2)	4	(0.5)			
Sometimes	300	(14.9)	209	(17.3)	91	(11.2)			
**Do you obey all government rules related to the COVID?**									
Yes	1784	(88.4)	1030	(85.4)	754	(93.0)	29.710	2	<0.001
No	39	(1.9)	25	(2.1)	14	(1.7)			
Sometimes	194	(9.6)	151	(12.5)	43	(5.3)			

Furthermore, 55.2% of respondents had more frequent practices towards ~~the~~ COVID-19. The rates of more frequent practices were significantly more likely to be (ⅰ)female (59.2% vs. 52.6% in male, *p*=.003), (ⅱ) older (age > 30 years) (64.0% vs. 48.6% in aged 12-20 years, *p*<.001), (ⅲ) have higher education (63.6% vs. 35.0% in secondary [6th-10th grades], *p*<.001), (ⅳ) be a housewife (68.8% vs. 52.2% in students, *p*=.001), (ⅴ) have monthly family income 20,000-30,000 BDT (57.9% vs. 48.6% in those < 20,000, *p*=.002), (ⅵ) be a respondent from urban area (58.7% vs. 47.2% in those from rural areas, *p*<.001), and (ⅶ) have more positive attitudes (58.6% vs. 49.7% in comparatively less positive attitudes, *p*<.001).

The sociodemographic factors of more frequent practices were sex (males vs females: OR=0.76; 95%CI=0.64-0.92, *p*=.003), being younger (12-20 years) vs. older (>30 years)(OR=0.53; 95%CI=0.38-0.75, *p*<.001), having secondary (6th-10th grades) vs. higher education(above bachelor) (OR=0.31; 95%CI=0.12-0.79, *p*=.014), having monthly family income less than 20,000 vs. more than 30,000 BDT (OR=0.71; 95%CI=0.57-0.88, *p*=.001), rural vs. urban area (OR=0.63; 95CI%=0.52-0.76, *p*<.001), and having more vs. comparatively less positive attitudes (OR=1.43; 95%CI=1.19-1.71, *p*<.001).

Discussion

This study was conducted aiming at measuring the level of knowledge, attitude, and practice of COVID-19 and perceptions regarding the disease among Bangladeshi people. The findings reveal a substantial number of sociodemographic factors that affect KAP and should prove useful when planning health education programs about emerging infectious diseases.

In the scope of perception towards COVID-19, the vast majority of the study participants reported some of the commonest symptoms of COVID-19 related [28], with only a very small minority being unaware of any of the symptoms, similar to other studies elsewhere [22,19]. Knowledge about the incubation period was also excellent and similar (86.2%) to the study conducted by Zegarra et al. [22] Similarly, routes of transmission of COVID-19 were reported by the participants: with only a minimal minority (0.2%) participants not being sure or unable of recognizing transmission routes. Perception of COVID-19 severity in the community showed that only 13.8 % did not face any difficulty when they discussed and tried to convince their family members about COVID-19 severity. Most of the responses a by the participants indicated negligence about the severity of the disease, reluctance to use masks, and the reluctance of complying with not being able to stop going out of the house. This may imply less participation in the preventive measures stipulated by the government as well as less inclination to observe social distancing and other individual preventive actions, although some alternative adaptive strategies were also mentioned. The most frequently identified gap in knowledge among participants was related to disease treatment. Only 18.3% of participants believed that there is no treatment for COVID-19, while 47.3% participants indicated that COVID-19 is a treatable disease, similar to another study [29]. Furthermore, only 1%of the participants reported vaccine as an option for preventing COVID-19, in marked contrast with

the previous study by Srichan et al which found that 31.2% were aware of the vaccine as a potential option [29]. In an earlier study by Aldowyan et al., only 19% of the participants were aware that there is no treatment for coronavirus like MERS-CoV, while 26.6% indicated the use of supportive treatment for MERS-CoV, and 31.1% of the participants mentioned the vaccine option for preventing MERS-CoV [24].

Compared to 3 others studies [17,29,30], our survey uncovered markedly reduced accurate knowledge, positive attitudes, and frequent practices towards COVID-19 [17,30]. This indicates a significant education gap, likely reflecting suboptimal public health information and dissemination regarding COVID-19, particularly since as indicted our survey primarily sampled educated younger people with ready access to a variety of information sources. Indeed, more accurate knowledge was significantly more likely among young adults, but intriguingly among respondents from rural areas, possibly reflecting that most of the participants were students, and that they all went back home, mostly to rural areas during the lockdown period. Srichanet al. found marital status, education, occupation, annual income were significant factors associated with accurate knowledge of COVID-19 [29], whereas Zhong et al. found that male sex, age-group of 16-29 years, marital status, education, employment and being a student were significantly associated with knowledge [17]. Therefore, tailoring of the information provided by health officials and other media outlets on the disease needs to address the multifactorial nature of the drivers leading to reduced knowledge.

The findings showed virtually universal agreement among the participants towards reporting to health authorities suspected cases of COVID-19, on ~~the issue~~ wearing a face mask before going to a crowded place, and in following other recommendations. These findings were similar to a very recent study conducted in China, during the rapid rise of COVID-19 outbreak

[17]. Saqlain et al. also reported positive attitudes among the vast majority of healthcare professionals towards wearing protective gear [29]. Similarly, the overall attitude towards actions such 'wash hands and face after coming from outside' and 'health education can play an important role for COVID-19 prevention' was universally favorable. Like in this study, Saqlain et al. reported that more than 80% participants strongly agreed that transmission of COVID-19 could be prevented by following universal precautions given by WHO or CDC [30]. During the SARS epidemic, 70.1-88.9% of Chinese residents believed that SARS can be successfully controlled or prevented [17,32]. Zhong et al. found that 90.8% of the respondents agreed that with control measures such as traffic limits all throughout China, and the shutdown of cities and counties of Hubei Province [17]. Surprisingly, the participants' attitudes differ by age, education, marital status, occupation, family type, monthly income, and practices. In contrast, Saqlain et al. found participants' attitudes were not affected by age, gender, experience, and job/occupation. Giao et al. also found that attitudes regarding COVID-19 did not present any significant associations with age, gender, and experience, but found a statistically significant association with occupation/job [32]. Also of relevance, Albarrak et al. and Khan et al. did not found any differences in attitude towards MERS among doctors, pharmacists, and nurses [33, 34].

In the multiple logistic regression analyses, sociodemographic variables associated with more positive attitudes regarding COVID-19 were older age, having higher education, being employed, having joint family, having higher monthly family income, and implementing more frequent practices, overall recapitulating previous findings from China [17].

The issue of preventive practices merits some comment since for some measures such as hand washing the results were remarkably similar to the findings others [30,34,35], albeit with the

exception of the study by Srichan et al., in which 54.8% did not regularly use soap during washing of hands [29]. Globally, women were significantly more likely to adopt preventive activities than men, a finding that may be of critical importance since targeting of women during household dissemination of education and preventive guidelines may ultimately yield improved implementation in households. Accordingly, we found that the sociodemographic factors associated with more frequent practice measures were being female, older age, having higher education, higher income, urban area residence, and having more positive attitudes. Male gender, occupation of "students", COVID-19 knowledge score, marital status, and residence were significantly associated factors in the Zhong et al. study, while experience was indicated by Saqlainet al., Hussain et al. and Ivey et al [30,36,37].

Considering the fact that Bangladesh is a multi-ethnic country with vastly different economic income, education levels, traditions, it is expected that the levels of knowledge, attitude, and prevention will also markedly differ in the population. Although good KAP was present in a sizeable proportion of the sample, it is very likely that population sectors that have no access to internet or live in regions with less likely fast escalation of transmission may also display reduced KAP when standard and uniform education and dissemination initiatives are promulgated and implemented. Indeed, it is highly probable that large clusters of people will become less informed and adoptive of prevention practices on COVID-19 [22]. Accessibility to information, dissemination and illustration of preventive behaviors, and sanitary educational measures are essential, especially in rural areas, among old people, poorer neighborhoods or communities, since these may have difficulties in getting access to novel information or encounter financial or resource barriers to implementation of preventive measures [15]. It is common consensus that a more educated population about any given disease will comply better with the preventive and treatment measures [38].

Limitations

This study has several limitations. First, this study followed a cross-sectional study design. Therefore, causal inferences may not be established. Second, compared with face-to-face interviews, self-reporting has limitations including multiple biases. Third, this study used an online-based survey method to avoid possible transmission, such that the cohort reflects sampling biases by being conducted online, thereby restricted to only those with internet access, and consequently unlikely to represent an accurate reflection of the whole Bangladeshi population. Notwithstanding, our study indicates that KAP assessments towards the COVID-19 pandemic of vulnerable populations warrant special effort to address the gaps incurred by the current study approach. Fourth, we used a limited number of questions to measure the level of knowledge, attitude, and practice. Thus, additional assessments would be important, using all aspects of KAP towards COVID-19, to determine the actual extent of KAP in the general population. Additionally, the unstandardized and inadequate assessment of attitudes and practices towards COVID should be developed via focus group discussion and in-depth interviews and constructed as multi-dimensional measures.

Conclusion

Our findings indicate that after the immediate lockdown and during the rapid rise period of the COVID-19 outbreak, internet users in Bangladesh displayed substantial differences in KAP regarding the pandemic. Our findings suggest the need for effective and tailored health education programs aimed at improving COVID-19 knowledge, thereby leading to more favorable attitudes and to implementation and maintenance of safe practices.

Acknowledgments

The authors appreciate all those who participated in this study voluntarily. Furthermore, the authors acknowledge the contributions and assistance of Rakib Hasan, Lakshmi Rani Kundu, A.S.M Mahbubul Alam, Gobida Deb Arya, Arza Miraz Keya, Md. Abdul Halim, Md Marzan Sarkar, Assaduzzaman Nur, Mohammad Yusuf, Jobair Sami, Md. Sanzid Mostofa, Miraz Mostafa, Mahmdul Hasan Shoron, Prokriti Biswas, Piya Ferdous, Mahir Shahariar Showrov, Sadman Sakib Samir, Maisha Meherin, Syeda Surayia Sultana, Sayma Islam Alin, Rejina Akter, A H Shourav, Kifayat Sadman Ishadi, Md. Safiul Hasan, Tasnima Akter Tasin, Fatema Akter Bethi, Sanzida Amin, Arpita Chakrabarty, Sayeda Sumaiya Nahrin, Rabeya Akter Mohua, Md. Rayhan Sakib, Tareq Mahmud, Md. Fakhrul Islam Maruf, Anik Roy, Tariqul Islam, Tasnimul Ahsan Shakhar, and Team SBCC (Social Behavior and Change Communication), during data collection periods.

References

1. Ruan S. Likelihood of survival of coronavirus disease 2019. Lancet Infect Dis. 2020;S1473-3099(20)30257-7. doi:10.1016/S1473-3099(20)30257-7
2. World Health Organization. 2019-nCoV outbreak is an emergency of international concern. 2020 [cited 2020 June 02]. Available from: http://www.euro.who.int/en/health-topics/health-emergencies/international-health-regulations/news/news/2020/2/2019-ncov-outbreak-is-an-emergency-of-international-concern.
3. World Health Organization. COVID-19 situation report no. #11. 2020 [cited 2020 June 02]. Available from: https://www.who.int/docs/default-source/searo/bangladesh/covid-19-who-bangladesh-situation-reports/who-ban-covid-19-sitrep-11.pdf?sfvrsn=ee79ca3d_6World.
4. Dhaka Tribune. 20-fold rise in Covid-19 cases in Bangladesh since April 1. 2020 [cited 2020 June 02]. Available from: https://www.dhakatribune.com/health/coronavirus/2020/04/14/20-fold-rise-of-covid-19-cases-in-bangladesh-since-april-1.
5. Roy D, Tripathy S, Kar SK, Sharma N, Verma SK, Kaushal V. Study of knowledge, attitude, anxiety & perceived mental healthcare need in Indian population during COVID-19 pandemic. Asian J Psychiatr. 2020;51:102083. doi:10.1016/j.ajp.2020.102083
6. Ilesanmi O, Alele FO. Knowledge, Attitude and Perception of Ebola Virus Disease among Secondary School Students in Ondo State, Nigeria, October, 2014. PLoS Curr. 2016;8:ecurrents.outbreaks.c04b88cd5cd03cccb99e125657eecd76. doi:10.1371/currents.outbreaks.c04b88cd5cd03cccb99e125657eecd76
7. BBC. Coronavirus: “Public holiday” extended in Bangladesh for 7 days, banned from going out after 6 pm,https://www.bbc.com/bengali/news-52241434; 2020 [accessed 16 May 2020]

8. Bdnews24.com.Bangladesh in virtual lockdown as coronavirus fight flares.2020 [cited 2020 June 02]. Available from: https://bdnews24.com/bangladesh/2020/03/25/bangladesh-in-virtual-lockdown-as-coronavirus-fight-flares.
9. Corona Info. Lockdown status. 2020 [cited 2020 June 02]. Available from: https://corona.gov.bd/lockdown-status.
10. AjiloreK, Atakiti I,Onyenankeya K. College students' knowledge, attitudes and adherence to public service announcements on Ebola in Nigeria: Suggestions for improving future Ebola prevention education programmes. Health Education Journal. 2017;76(6):648-660. doi:10.1177/0017896917710969
11. Tachfouti N, Slama K, Berraho M, Nejjari C. The impact of knowledge and attitudes on adherence to tuberculosis treatment: a case-control study in a Moroccan region. Pan Afr Med J. 2012;12:52.
12. TaoN.An analysis on reasons of SARS-induced psychological panic among students. Journal of Anhui Institute of Education. 2013;21:78-79.
13. Person B, Sy F, Holton K, Govert B, Liang A; National Center for Inectious Diseases/SARS Community Outreach Team. Fear and stigma: the epidemic within the SARS outbreak. Emerg Infect Dis. 2004;10(2):358- 363. doi:10.3201/eid1002.030750
14. Bewick S, Fagan WF, Calabrese JM, Agusto F.Zika virus: Endemic versus epidemic dynamics and implications for disease spread in the Americas. BioRxiv. 2016;41897.
15. Szymona-Pałkowska K, Janowski K, Pedrycz A, et al. Knowledge of the Disease, Perceived Social Support, and Cognitive Appraisals in Women with Urinary Incontinence. Biomed Res Int. 2016;2016:3694792. doi:10.1155/2016/3694792

16. Zhou M, Tang F, Wang Y, et al. Knowledge, attitude and practice regarding COVID-19 among health care workers in Henan, China. J Hosp Infect. 2020;S0195-6701(20)30187-0. doi:10.1016/j.jhin.2020.04.012 18.

17. Zhong BL, Luo W, Li HM, et al. Knowledge, attitudes, and practices towards COVID-19 among Chinese residents during the rapid rise period of the COVID-19 outbreak: a quick online cross-sectional survey. Int J Biol Sci. 2020;16(10):1745- 1752. doi:10.7150/ijbs.45221

18. Corrin T, Waddell L, Greig J, Young I, Hierlihy C, Mascarenhas M. Risk perceptions, attitudes, and knowledge of chikungunya among the public and health professionals: a systematic review. Trop Med Health. 2017;45:21. doi:10.1186/s41182-017-0061-x

19. Janjua NZ, Razaq M, Chandir S, Rozi S, Mahmood B. Poor knowledge--predictor of nonadherence to universal precautions for blood borne pathogens at first level care facilities in Pakistan. BMC Infect Dis. 2007;7:81. doi:10.1186/1471-2334-7-81

20. Lau JT, Kim JH, Tsui H, Griffiths S. Perceptions related to human avian influenza and their associations with anticipated psychological and behavioral responses at the onset of outbreak in the Hong Kong Chinese general population. Am J Infect Control. 2007;35(1):38- 49. doi:10.1016/j.ajic.2006.07.010

21. Smith RD. Responding to global infectious disease outbreaks: lessons from SARS on the role of risk perception, communication and management. Soc Sci Med. 2006;63(12):3113- 3123. doi:10.1016/j.socscimed.2006.08.004

22. ZegarraA, Chino B, Ames R. Knowledge, perception and attitudes in Regard to COVID-19. Pandemic in Peruvian Population. 2020. doi:10.31234/osf.io/kr9ya

23. ALdowyan N, Abdallah A, & El-Gharbawy R. (2017). Knowledge, Attitude and Practice (KAP) Study about Middle East Respiratory Syndrome Coronavirus (MERS-CoV) among Population in Saudi Arabia. International Archives of Medicine, 10. doi:10.3823/2524

24. Medani KET, ALDuwayhis NM, ALAmeer AA, ALMaymoni SK, ALOtaibiFA, ALHassan MA. Knowledge, Attitude and Practice of Middle East Respiratory Syndrome Corona Virus (Mers- CoV), Among Male Primary School Students in Almajmaah City, Saudi Arabia. Indo Am. J. P. Sci. 2018;5(12):6288-16296. doi:0.5281/zenodo.2362589
25. World Health Organization. Coronavirus disease (COVID-19) advice for the public: Myth busters. 2020 [cited 2020 June 02]. Available from: https://www.who.int/emergencies/diseases/novel-coronavirus-2019/advice-for-public/myth-busters.
26. Institute of Epidemiology Disease Control and Research (IEDCR). Interim infection prevention and control recommendations for suspected/confirmed patients with 2019 Novel coronavirus (2019-nCoV) in healthcare settings. 2020 [cited 2020 June 02]. Available from: https://www.iedcr.gov.bd/website/images/files/nCoV/Infection Prevention and Control Recommendations_v2.pdf.
27. TaberKS. The use of Cronbach's alpha when developing and reporting research instruments in science education. Res Sci Educ, 2018;48(6):1273-1296. doi:10.1007/s11165-016-9602-2
28. Biskup E, Chen G, Ferretti M. Understanding COVID-19 new diagnostic guidelines – A message of reassurance from an internal medicine doctor in Shanghai. Swiss Med Wkly. 2020;150:w20216. doi:10.4414/smw.2020.20216
29. Srichan P, Apidechkul T, Tamornpark R, Yeemard F, Khunthason S, Kitchanapaiboon S, Wongnuch P, Wongphaet A, Upala P. Knowledge, Attitude and Preparedness to Respond to the 2019 Novel Coronavirus (COVID-19) Among the Bordered Population of Northern Thailand in the Early Period of the Outbreak: A Cross-Sectional Study. SSRN Electronic Journal. 2020. doi:10.2139/ssrn.3546046

30. Saqlain M, Munir MM, Ur Rehman S, Gulzar A, Naz S, Ahmed Z, Tahir AH, Mashhood M. Knowledge, attitude, practice and perceived barriers among healthcare professionals regarding COVID-19: A Cross-sectional survey from Pakistan. medRxiv, 2020. doi: 10.1101/2020.04.13.20063198
31. Adhikari SP, Meng S, Wu YJ, et al. Epidemiology, causes, clinical manifestation and diagnosis, prevention and control of coronavirus disease (COVID-19) during the early outbreak period: a scoping review. Infect Dis Poverty. 2020;9(1):29. doi:10.1186/s40249-020-00646-x
32. Giao H, Nguyen TNH, Tran VK, Ngan Vo K, NganVK, Tam V, Le AnP. Knowledge and attitude toward COVID-19 among healthcare workers at District 2 Hospital, Ho Chi Minh City. Asian Pacific Journal of Tropical Medicine. 2020. doi:10.4103/1995-7645.280396
33. Albarrak AI, Mohammed R, Al Elayan A, et al. Middle East Respiratory Syndrome (MERS): Comparing the knowledge, attitude and practices of different health care workers. J Infect Public Health. 2019;S1876-0341(19)30239-4. doi:10.1016/j.jiph.2019.06.029
34. Khan MU, Shah S, Ahmad A, Fatokun O. Knowledge and attitude of healthcare workers about Middle East Respiratory Syndrome in multispecialty hospitals of Qassim, Saudi Arabia. BMC Public Health. 2014;14:1281. doi:10.1186/1471-2458-14-1281
35. Nour M, Babilghith A, Natto H, Elamin F, Alawneh S. Knowledge, attitude and practices of healthcare providers towards MERS-CoV infection at Makkah hospitals, KSA. International Research Journal of Medicine and Medical Sciences. 2015;3:103-112.
36. Ivey MF. Global opportunity: Pharmacists working together to improve patient care. Am J Health Syst Pharm. 2019;76(12):869-872. doi:10.1093/ajhp/zxz071
37. Hussain, R, Hassali, MA, Hashmi F, Farooqui M. A qualitative exploration of knowledge, attitudes and practices of hospital pharmacists towards adverse drug reaction reporting

system in Lahore, Pakistan. Journal of Pharmaceutical Policy and Practice. 2018;11:16. doi:10.1186/s40545-018-0143-0

38. Hocking A, Laurence C, Lorimer M. Patients' knowledge of their chronic disease: The influence of socio-demographic characteristics. Australian Family Physician. 2013;42(6):411.

Table 6. Distribution and risk factors of Knowledge, Attitude and Practice among participants

Variables	Knowledge								Attitudes								Practices							
	Less accurate		More accurate		χ[2] *p-value*	OR	95%CI	*p-value*	Less positive		More positive		χ[2] *p-value*	OR	95%CI	*p-value*	Less frequent		More frequent		χ[2] *p-value*	OR	95%CI	*p-value*
	N	(%)	N	(%)					N	(%)	N	(%)					N	(%)	N	(%)				
**Gender**																								
Male	626	(51.9)	580	(48.1)	0.046	0.981	0.821-1.172	0.829	472	(39.1)	734	(60.9)	2.715	0.856	0.712-1.030	0.100	572	(47.4)	634	(52.6)	8.583	0.764	0.638-0.915	0.003
Female	417	(51.4)	394	(48.6)	0.829	1			288	(35.5)	523	(64.5)	0.099	1			331	(40.8)	480	(59.2)	0.003	1		
**Age (years)**																								
12-20	340	(50.7)	331	(49.3)	7.100	1.538	1.098-2.155	0.012	301	(44.9)	370	(55.1)	25.841	0.467	0.325-.671	<0.001	345	(51.4)	326	(48.6)	20.474	0.530	0.377-0.746	<0.001
21-30	594	(50.9)	574	(49.1)	0.029	1.527	1.106-2.108	0.010	410	(35.1)	758	(64.9)	<0.001	0.702	0.495-.997	0.048	494	(42.3)	674	(57.7)	<0.001	0.766	0.552-1.063	0.110
>30	109	(61.2)	69	(38.8)		1			49	(27.5)	129	(72.5)		1			64	(36.0)	114	(64.0)		1		
**Education**																								
Secondary (6-10)	8	(40.0)	12	(60.0)	4.347	1.419	0.571-3.527	0.451	9	(45.0)	11	(55.0)	47.791	0.427	0.173-1.051	0.064	13	(65.0)	7	(35.0)	23.415	0.308	0.121-0.786	0.014
Intermediate (11-12)	117	(51.8)	109	(48.2)	0.226	0.881	0.646-1.202	0.426	108	(47.8)	118	(52.2)	<0.001	0.381	0.276-0.528	<0.001	110	(48.7)	116	(51.3)	<0.001	0.604	0.441-0.827	0.002
Bachelor	655	(53.3)	575	(46.7)		0.830	0.678-1.017	0.072	503	(40.9)	727	(59.1)		0.505	0.404-0.631	<0.001	583	(47.4)	647	(52.6)		0.636	0.516-0.782	<0.001
Higher education (above bachelor)	263	(48.6)	278	(51.4)		1			140	(25.9)	401	(74.1)		1			197	(36.4)	344	(63.6)		1		
**Marital status**																								
Single	835	(51.2)	795	(48.8)	1.659	0.571	0.136-2.398	0.444	643	(39.4)	987	(60.6)	14.932	2.558	0.609-10.742	0.199	751	(46.1)	879	(53.9)	5.854	0.702	0.167-2.948	0.629
Married	205	(54.1)	174	(45.9)	0.436	0.509	0.120-2.161	0.360	112	(29.6)	267	(70.4)	0.001	3.973	0.934-16.909	0.062	149	(39.3)	230	(60.7)	0.054	0.926	0.218-3.933	0.917
Divorced	3	(37.5)	5	(62.5)		1			5	(62.5)	3	(37.5)		1			3	(37.5)	5	(62.5)		1		
**Occupation**																								
Student	733	(51.0)	704	(49.0)	5.584	1.633	0.743-3.590	0.223	601	(41.8)	836	(58.2)	40.508	0.397	0.159-0.991	0.048	687	(47.8)	750	(52.2)	20.203	0.873	0.406-1.879	0.729
Housewife	40	(62.5)	24	(37.5)	0.349	1.020	0.402-2.587	0.967	14	(21.9)	50	(78.1)	<0.001	1.020	0.345-3.016	0.971	20	(31.3)	44	(68.8)	0.001	1.760	0.698-4.438	0.231
Govt. employee	59	(48.4)	63	(51.6)		1.815	0.770-4.281	0.173	38	(31.1)	84	(68.9)		0.632	0.236-1.691	0.360	46	(37.7)	76	(62.3)		1.322	0.569-3.070	0.517
Non-govt. employee	165	(52.4)	150	(47.6)		1.545	0.686-3.480	0.293	82	(26.0)	233	(74.0)		0.812	0.317-2.082	0.664	118	(37.5)	197	(62.5)		1.336	0.605-2.951	0.474
Businessman	29	(55.8)	23	(44.2)		1.348	0.519-3.499	0.539	19	(36.5)	33	(63.5)		0.496	0.170-1.445	0.199	20	(38.5)	32	(61.5)		1.280	0.499-3.285	0.608
Unemployedt	17	(63.0)	10	(37.0)		1			6	(22.2)	21	(77.8)		1			12	(44.4)	15	(55.6)		1		
**Family type**																								
Nuclear	809	(51.5)	763	(48.5)	0.175	1.046	0.847-1.291	0.676	612	(38.9)	960	(61.1)	4.753	0.782	0.626-0.976	0.029	707	(45.0)	865	(55.0)	0.121	0.963	0.779-1.190	0.728
Joint	234	(52.6)	211	(47.4)	0.676	1			148	(33.3)	297	(66.7)	0.029	1			196	(44.0)	249	(56.0)	0.728	1		
**No of family member**																								
<5	746	(51.5)	703	(48.5)	0.106	1.033	0.850-1.254	0.745	553	(38.2)	896	(61.8)	0.514	0.929	0.760-1.136	0.473	631	(43.5)	818	(56.5)	3.108	1.191	0.981-1.447	0.078
≥5	297	(52.3)	271	(47.7)	0.745	1			207	(36.4)	361	(63.6)	0.473	1			272	(47.9)	296	(52.1)	0.078	1		
**Monthly family income**																								
<20,000	256	(50.0)	256	(50.0)	1.412	1.066	0.861-1.318	0.559	216	(42.2)	296	(57.8)	8.219	0.732	0.589-0.911	0.005	263	(51.4)	249	(48.6)	12.147	0.707	0.571-0.876	0.001
20,000-30,000	267	(53.7)	230	(46.3)	0.494	0.918	0.740-1.138	0.436	193	(38.8)	304	(61.2)	0.016	0.842	0.674-1.051	0.128	209	(42.1)	288	(57.9)	0.002	1.029	0.828-1.279	0.795
>30,000	520	(51.6)	488	(48.4)		1			351	(34.8)	657	(65.2)		1			431	(42.8)	577	(57.2)		1		
**Residence**																								
Rural area	288	(47.2)	322	(52.8)	7.083	1.295	1.070-1.566	0.008	238	(39.0)	372	(61.0)	0.665	0.922	0.758-1.121	0.415	322	(52.8)	288	(47.2)	22.733	0.629	0.520-0.762	<0.001
Urban area	755	(53.7)	652	(46.3)	0.008	1			522	(37.1)	885	(62.9)	0.415	1			581	(41.3)	826	(58.7)	<0.001	1		
**Knowledge**																								
More accurate	0	(.0)	974	(100.0)	—	—	—	—	372	(38.2)	602	(61.8)	0.211	0.959	0.801-1.148	0.646	424	(43.5)	550	(56.5)	1.167	1.102	0.924-1.313	0.280
Less accurate	1043	(100.0)	0	(0.0)	—				388	(37.2)	655	(62.8)	0.646	1			479	(45.9)	564	(54.1)	0.280	1		
**Attitude**																								
More positive	655	(52.1)	602	(47.9)	0.211	0.959	0.801-1.148	0.646	0	(0.0)	1257	(100.0)	—	—	—	—	521	(41.4)	736	(58.6)	14.885	1.428	1.191-1.711	<0.001
Less Positive	388	(51.1)	372	(48.9)	0.646	1			760	(100.0)	0	(0.0)	—				382	(50.3)	378	(49.7)	<0.001	1		
**Practice**																								
More frequent	564	(50.6)	550	(49.4)	1.167	1.102	0.924-1.313	0.280	378	(33.9)	736	(66.1)	14.885	1.428	1.191-1.711	<0.001	0	(0.0)	1114	(100.0)	—	—	—	—
Less frequent	479	(53.0)	424	(47.0)	0.280	1			382	(42.3)	521	(57.7)	<0.001	1			903	(100.0)	0	(0.0)	—			
