# Supplementary material for: Knowledge, attitude, and practice regarding COVID-19 outbreak in Bangladesh: An online-based cross-sectional study
Source: PLoS One. 2020 Oct 9;15(10):e0239254. doi: 10.1371/journal.pone.0239254 (PMC7546509; doi:10.1371/journal.pone.0239254)
Supplement: Attachment — Submitted filename: PLOS One Response to Reviewers 2020.docx [file pone.0239254.s003.docx]

Dear Editor,

We would like to thank you for your consideration of the manuscript ‘Knowledge, attitude, and practice regarding COVID-19 outbreak in Bangladesh: An online-based cross-sectional study’ and for the feedback provided by the two reviewers. We have addressed their comments, and revised the manuscript accordingly. We are resubmitting the manuscript and hope that the paper is now acceptable for publication in *PLOS ONE.*

**Additional comments**

**a)** If there are ethical or legal restrictions on sharing a de-identified data set, please explain them in detail (e.g., data contain potentially identifying or sensitive patient information) and who has imposed them (e.g., an ethics committee). Please also provide contact information for a data access committee, ethics committee, or other institutional body to which data requests may be sent.

***Authors’ response:*** The anonymized dataset has been uploaded.

**b)** If there are no restrictions, please upload the minimal anonymized data set necessary to replicate your study findings as either Supporting Information files or to a stable, public repository and provide us with the relevant URLs, DOIs, or accession numbers. Please see http://www.bmj.com/content/340/bmj.c181.long for guidelines on how to de-identify and prepare clinical data for publication. For a list of acceptable repositories, please see <http://journals.plos.org/plosone/s/data-availability#loc-recommended-repositories>.

***Authors’ response:*** The anonymized dataset has been uploaded.

**Responses to Reviewer 1**

***Reviewer’s comment:*** The work presented meets the requirements for publication, I would expect to find more information about the instrument's construction and validation process. If it were the case, the qualitative validation.

***Authors’ response:*** Thank you for the positive feedback. We have now discussed this issue in Methods section regarding instrument's construction and validation process.

**Responses to Reviewer 2**

***Reviewer’s comment:*** Manuscript needs to be checked for grammar/language (highlighted in file attached).

***Authors’ response:*** All suggested changes are implemented in the revised version of manuscript.

***Reviewer’s comment:*** Knowledge, attitude, practice (page 6): Cronbach alpha must be calculated separately for knowledge, attitudes and practice. Also calculated and reported for pilot data and field data.

***Authors’ response:*** This has been done as requested and added in Methods section.

***Reviewer’s comment:*** Knowledge, attitude, practice (page 7): How was “dont know” coded for the knowledge items? How was the total score for practices calculated?

***Authors’ response:*** The specifics are stated clearly in Methods section.

***Reviewer’s comment:*** Perception (page 8): Perceptions between men and women were presented in Table 2. Why was this important to observe/report?

***Authors’ response:*** Sex differences are always important to consider; for example, please note the differences in mask adherence between women and men during COVID-19 (see for example: <https://psyarxiv.com/tg7vz>.)

***Reviewer’s comment:*** Knowledge (page 12): Accurate, adequate, inaccurate knowledge scores - what score ranges indicate these categories?

***Authors’ response:*** It was supposed to be more accurate knowledge and apologize for the mistake. Because there is no standard cutoff to determine accurate or adequate or inaccurate knowledge towards the COVID-19, we treated this as linear continuous data, and accordingly mentioned ‘*more accurate knowledge*’ term.

***Reviewer’s comment:*** Practice (page 15): Reference the table for binary logistic regression of practice (Table 6) in the write-up.

***Authors’ response:*** Previously the reference category was coded as “1” in Table 6. Now it has been replaced “1” as “Ref.”.

***Reviewer’s comment:*** Any ideas as to why positive attitudes were significantly more likely to be older, married, housewives, joint family etc?

***Authors’ response:*** We can only infer that such findings reflect family stability and previous experience, and have included a comment to this effect in the Discussion

***Reviewer’s comment:*** Also any potential reasons why more frequent practices among males, younger people, lower income etc?

***Authors’ response:*** We believe that the Reviewer misunderstood our findings; the OR were <1 indicating that females, older etc… were more likely to implement preventive practices. We can only infer that such findings reflect intrinsic sex differences as well as other socioeconomic factors (see for example: <https://psyarxiv.com/tg7vz>.)
